# Inflammatory Regulation of CNS Barriers After Traumatic Brain Injury: A Tale Directed by Interleukin-1

**DOI:** 10.3389/fimmu.2021.688254

**Published:** 2021-05-21

**Authors:** Colleen N. Bodnar, James B. Watson, Emma K. Higgins, Ning Quan, Adam D. Bachstetter

**Affiliations:** ^1^ Department of Neuroscience, University of Kentucky, Lexington, KY, United States; ^2^ Spinal Cord and Brain Injury Research Center, University of Kentucky, Lexington, KY, United States; ^3^ Department of Biomedical Science, Charles E. Schmidt College of Medicine and Brain Institute, Florida Atlantic University, Jupiter, FL, United States

**Keywords:** neuroinflammation, neuroimmunology, glia, neurotrauma, edema, interleukin-1 receptor 1, IL1R1

## Abstract

Several barriers separate the central nervous system (CNS) from the rest of the body. These barriers are essential for regulating the movement of fluid, ions, molecules, and immune cells into and out of the brain parenchyma. Each CNS barrier is unique and highly dynamic. Endothelial cells, epithelial cells, pericytes, astrocytes, and other cellular constituents each have intricate functions that are essential to sustain the brain’s health. Along with damaging neurons, a traumatic brain injury (TBI) also directly insults the CNS barrier-forming cells. Disruption to the barriers first occurs by physical damage to the cells, called the primary injury. Subsequently, during the secondary injury cascade, a further array of molecular and biochemical changes occurs at the barriers. These changes are focused on rebuilding and remodeling, as well as movement of immune cells and waste into and out of the brain. Secondary injury cascades further damage the CNS barriers. Inflammation is central to healthy remodeling of CNS barriers. However, inflammation, as a secondary pathology, also plays a role in the chronic disruption of the barriers’ functions after TBI. The goal of this paper is to review the different barriers of the brain, including (1) the blood-brain barrier, (2) the blood-cerebrospinal fluid barrier, (3) the meningeal barrier, (4) the blood-retina barrier, and (5) the brain-lesion border. We then detail the changes at these barriers due to both primary and secondary injury following TBI and indicate areas open for future research and discoveries. Finally, we describe the unique function of the pro-inflammatory cytokine interleukin-1 as a central actor in the inflammatory regulation of CNS barrier function and dysfunction after a TBI.

## Introduction

The central nervous system (CNS) relies on the immune system for proper maintenance, recovery, and repair functions in health, disease, or following neurotrauma. Yet, the CNS and the vast majority of the systemic immune system are separated by various barriers (1). The CNS barriers – often erroneously thought of as solid fortress walls separating the CNS from the body – are highly dynamic. Extensive cellular and molecular interactions coordinate the ingress and egress of fluid, molecules, and cells across the CNS borders. A pivotal point in the loss of healthy CNS function often occurs when the coordination of events at the CNS barriers is skewed. There are many examples of barrier disruption in CNS diseases. There may be no better example of barrier disruption than neurotrauma.

Each year, an estimated 2 million people in the United States alone sustain a TBI (2). While these numbers are significant, they still vastly underestimate the pervasiveness of TBIs. Many individuals, including victims of intimate partner violence, fail to report their injuries, while others may not need or seek emergency medical treatment (2, 3). The damage caused to the brain, regardless of hospitalization, is real. A TBI, including one that may be viewed as ‘only a concussion,’ can have lasting impacts on one’s quality of life.

The physical disruption of the cells within the brain due to the force of a TBI constitute the primary injury and can affect neurons, glia and vasculature (4). The primary injury then starts secondary injury cascades, which can disrupt the brain and its barriers for months to years after the initial injury (5). These secondary injury cascades involve many biochemical and cellular events that seek to restore brain function and worsen the neural damage caused by the primary injury.

Secondary injury mechanisms are those processes most amenable to therapeutic intervention. Following injury anywhere in the body, control of inflammation is one of the most clinically successful ways to restore function and quality of life – although the CNS has largely been left out of these successful treatments. After a TBI, inflammation in the brain is different from inflammation to peripheral tissues. The CNS barriers are largely responsible for these differences.

Interleukin-1 (IL-1) is a ‘master cytokine,’ initiating a cascade of inflammatory pathways. IL-1 exists in two forms IL-1α and IL-1β. Both IL-1α and IL-1β signal mainly through IL-1 Receptor 1 (IL-1R1) (6). IL-1R1 is uniquely positioned at critical CNS barrier interfaces (7–9). Further, IL-1 can remain chronically above normal levels for months to years after TBI (10, 11). Thus, IL-1/IL-1R1 is likely a critical control hub for restoring CNS barriers after an injury.

In the current review, we will present the different CNS barriers and describe how both primary and secondary injury after TBI changes these barriers. We will then explain how inflammation, explicitly driven by pro-inflammatory cytokine interleukin-1 (IL-1), might be a mechanism behind changes to CNS barriers following TBI. This review will highlight the essential and dynamic role of CNS barriers in TBI-secondary injury cascades and describe how, through the modulation of IL-1, therapies can be developed to help people recover and do so more quickly after a TBI and return to their preinjury life.

## Blood Brain Barrier (BBB)

Injection of trypan blue dye into the vasculature, which stained the vasculature throughout the body, yet left the CNS unmarked, led to the BBB discovery over a century ago. In the subsequent 100+ years, many illustrative studies have defined the vital and dynamic nature of the BBB in health and disease. While it is well known that a TBI disrupts the BBB, there is still much that remains unexplored, both in terms of the natural history of BBB disruption as a contributor to TBI-induced dysfunction and as a viable therapeutic target.

### Blood Brain Barrier Overview

**Figure 1:**
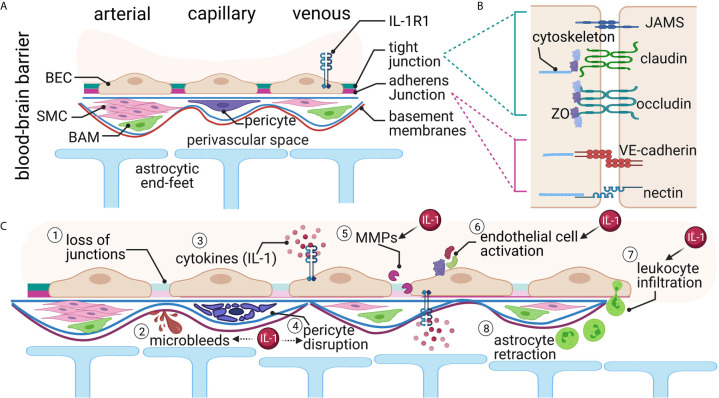
The Blood Brain Barrier (BBB). **(A)** Brain endothelial cells (BEC) form the first layer of the BBB. An endothelial basement membrane then surrounds the BECs. Smooth muscle cells (SMC), pericytes, and border-associated macrophages (BAM) are in the next layer. Finally, the parenchymal basement membrane and the astrocytic end-feet create the perivascular space. Interleukin-1 receptor 1 (IL-1R1) is expressed in the venous compartment of the BECs, presumably on both the luminal and abluminal sides. **(B)** The junctions between endothelial cells form the major barrier component and consist of several proteins both in the intercellular space and intracellular compartments. **(C)** Traumatic brain injury causes several alterations at the BBB. These changes include (1) loss of junctional proteins, (2) microbleeds, (3) local and systemic increased cytokines, (4) pericyte disruption, (5) matrix metalloprotease (MMP) activation, (6) endothelial cell activation, (7) leukocyte infiltration, and (8) astrocyte retraction from the barrier. Many of these changes have been linked either directly (solid arrow) or indirectly (dashed arrow) with IL-1 signaling.

The BBB is a highly regulated physiologic barrier between the CNS and the periphery, with specialized brain endothelial cells controlling the majority of the barrier functions (**Figure 1A**). The brain endothelial cells block the paracellular diffusion of water-soluble molecules into the brain parenchyma. At the same time, blood gases, oxygen and carbon dioxide can diffuse passively across the BBB, as well as many small, lipophilic molecules (12, 13). However, larger polar molecules and many essential nutrients, such as glucose and amino acids, cannot diffuse across the BBB and are blocked from passing through the paracellular route. Instead, to traverse the BBB, brain endothelial cells use selective transporters and channels to move more complex molecules like essential nutrients, ions, and other polar, hydrophilic molecules across the BBB (12, 13). Specific methods of transport include solute carriers, ATP-binding cassette transporters, receptor mediated transcytosis, and mononuclear cell migration (12).

The tight junctions and adherens junctions exist between brain endothelial cells to create a tightly-sealed barrier. Tight junctions are located apically between endothelial cells and are arranged in strands of interconnected, intramembranous, integral membrane proteins (14, 15) (**Figure 1B**). The primary components of tight junctions in the BBB are occludins, claudins, junctional adhesion molecules (JAMS), zonula occludens (ZO) proteins, and cytoplasmic accessory proteins (16, 17). Adherens junctions, located in the paracellular space near the endothelial cells’ basement membrane, significantly influence functional tight junctions (**Figure 1B**). Both adherens junctions and tight junctions are highly influenced by each other (12, 17–19). The main component of adherens junctions is cadherins, which serve the role of mediating cell-cell, homophilic adhesion between adjacent endothelial cells (18–20). Together, these proteins restrict the paracellular pathway into the CNS and impart the high electrical resistance characteristic to the BBB (12, 21).

While the brain endothelial cells are integral to the creation of a tight BBB, many other cells play active roles in the function of the BBB (**Figure 1A**). The BBB contains a second basement membrane, the parenchymal basement membrane, where astrocytic end-feet tether (18). The astrocytic end-feet surround 98% of the CNS microvasculature (22). Together with the parenchymal basement membrane, astrocytes compose the glial limitans, which block the passage of leukocytes into the CNS, thus contributing to the immune privilege of the CNS (18, 23). Additionally, pericytes play a vital role in maintaining the BBB’s integrity. Pericytes block larger molecules and ensure the astrocytic end-feet’s polarization, regulating the astrocytic coupling to the BBB (24, 25).

Macrophages associated with the BBB (termed border-associated macrophages - BAMs) are found in the perivascular space and other barriers, including the blood-CSF barrier and the meninges (26). These cells are critical in phagocytosis and innate immune regulation and surveillance and have similar functions to the microglia in the brain parenchyma (26).

### Blood Brain Barrier Disruption After TBI

The cells of the BBB can withstand a certain amount of mechanical strain. However, with TBI, this amount of tolerable pressure is exceeded, resulting in the physical breaking of the barrier (4). These mechanical changes result in bleeding, shearing of tight junctions, and lesions to endothelial cells, astrocytes, and pericytes following TBI (**Figure 1C**). Secondary cascades continue for days-to-weeks after the injury, further disrupting the BBB.

Evidence of bloody lesions in the brain on neuroimaging (CT or MRI) is used clinically to assess the TBI severity. At all severity levels of TBI, bleeds occur in animal models and human patients (27), which may not be visible by routine clinical CT methods, but can be seen by MRI and microscopically (**Figure 1C**). For instance, small petechial hemorrhages have been found in both children *via* T2 weighted MRI (28) and adults *via* susceptibility-weighted MRI (29). In humans, these small microbleeds occur throughout the brain after TBI but specifically accumulate at the brain’s more physically vulnerable areas, such as the white/gray matter interfaces (30). Microbleeds are a prevalent pathology following mild (27%), moderate (47%), and severe (58%) TBI *via* T2 weighted MRI (27). Evidence of microbleeds is often the most prevalent pathology seen on imaging in human fluid-attenuation inversion recovery (FLAIR) MRI and can last up to 5 years after the initial injury, and those with microbleeds were two times more likely to have a long-term disability (31).

Junctional proteins get torn away from each other following TBI (**Figure 1C**). Occludin, claudins, and ZO-1, and cadherins, the major components of the BBB tight junctions, are reduced after rodent blast injury (32), rodent and pig controlled cortical impact (CCI) (33, 34), and cryogenic TBI (35). Shearing of tight junctions and lesions to endothelial cells increases leakage of blood serum proteins and other molecules across the BBB, as recently reviewed (36). Tearing of junctional proteins leads to the first phase of increased BBB permeability following TBI. When exposed to blood-derived factors, the BBB cells, including astrocytes and pericytes, become reactive. The second phase of increased BBB permeability after TBI is the result of biochemical changes as opposed to the physical disruption that dominates the primary injury-induced BBB permeability (37). Increased permeability of the BBB *via* these different mechanisms leads to increased fluid and edema formation (38, 39).

When brain endothelial cells become reactive from the primary and secondary injury (**Figure 1C**), there is increased permeability, hypertrophy, and hemostatic pathway activation (40). An example of alteration of brain endothelial cell function is disrupted transport. Typically, this is a highly regulated process. However, after TBI, there is an increase in transendothelial vacuolar and vesicular transport and increased pinocytotic activity (41). Lesions to brain endothelial cells following experimental TBI to rodents also increase permeability and further damage to the BBB. These changes increase intracranial pressure and edema formation (42).

Pericytes are also damaged following TBI (**Figure 1C**). For example, up to 40% of pericytes were found to be moved from the blood vessels after a TBI, as a result of the primary insult and secondary injury events (43). Further, TBI-induces morphological changes to pericytes including degeneration, necrosis, phagocytosis and hypertrophy (44, 45). This damage in pericytes has functional consequences including alterations in neurovascular coupling (46), which can lead to further disruption of the BBB.

Gene expression changes in BBB cells include alterations to various pathways that enhance inflammation, including major histocompatibility (MHC) class I and II molecules, E-selectin, VCAM-1 and ICAM-1 (40, 47). Also, matrix metalloproteinases (MMPs) and vascular endothelial growth factor (VEGF) are increased and linked to secondary barrier permeability after TBI. VEGF is a trophic factor that leads to increased angiogenesis (48), and increases blood vessels’ permeability as part of its angiogenic properties. A TBI increases both VEGF and its receptor (VEGFR) (49–51). VEGF causes phosphorylation of endothelial proteins like ZO-1 and VE-cadherin, resulting in loss of normal localization of these proteins (50). In microdialysate from severe human TBI, MMP-1, -2, -3, and -9 were increased (52). Similarly, TBI in rodents and cats increased MMP-2 and -9 (53–56). Microglia, astrocytes, pericytes and neurons can release MMPs (57, 58). Changes in MMP activity can also be activated by a loss of junctional proteins after experimental TBI (33). MMPs, once activated, attack basal lamina proteins and degrade tight junction proteins resulting in edema formation.

With the increased permeability of the BBB and the increased expression of vascular adhesion molecules on brain endothelial cells, there is increased leukocyte infiltration into the brain after a TBI (**Figure 1C**). Within hours-to-days after injury, leukocytes infiltrate into the brain by a process that requires interaction with adhesion proteins in the brain endothelial cells (i.e., selectins) and anchoring and docking proteins (i.e., ICAM-1) (50, 59). After passing through the brain endothelial cell barrier, the leukocytes must then pass the glial limitans. As the astrocytic end-feet are retracted from the BBB, early following a TBI, the leukocytes accumulate in the perivascular spaces (23), and tend to congregate, especially around venules (60).

Once in the brain, leukocytes increase the inflammation and further increase permeability, leading to edema (59, 61). For example, neutrophils release MMP-9 to aid in the additional recruitment of leukocytes into the brain (62), which further adds to the BBB breakdown, exacerbating neuroinflammation, neuronal degeneration, edema, and brain tissue damage (61). Experimentally blocking the recruitment of neutrophils to the injured brain reduced edema, tissue loss, apoptosis, and microglia number after rodent TBI (59, 63). The damaged caused by neutrophils – which peaks around 12-to-48 hours after experimental TBI (64) – occurs *via* reactive oxygen species, protease activation, elastase and MMP activation, and further pro-inflammatory cytokine release (59). In addition, when peripheral macrophages are blocked from entering the brain through antagonism of CCR2 after TBI, there is a reduction in the inflammatory profile as well as cognitive improvement (65). On the other hand, leukocyte infiltration into the brain parenchyma can be protective if disruption of the barriers allowed pathogens to enter the brain, for instance (66).

While not extensively studied at the BBB following TBI, border-associated macrophages are expected to become reactive like other macrophages (67). In ischemic stroke, border-associated macrophages increase vascular permeability, increasing cells’ ability to infiltrate from the periphery, which is correlated with reduced neurological function in stroke models (67). In a middle cerebral artery occlusion model, increased border-associated macrophages were seen in the perivascular and subpial spaces as early as 16 hours after insult (67). Further, these border-associated macrophages were characterized *via* RNA sequencing and had increased chemokine production for the recruitment of leukocytes across the BBB (67). As with other disease mechanisms, there is potentially a similar mechanism occurring following TBI, which has yet to be elucidated.

### Mechanisms of IL-1 Signaling at the Blood Brain Barrier After TBI

Two forms of IL-1 exist in the brain, IL-1α, and IL-1β. IL-1α is expressed constitutively and is considered a damage-associated molecular pattern. Microglia are the primary source of IL-1 (68), although all CNS cells and cells throughout the body can make IL-1. IL-1β is induced upon activation and then processed into the active form *via* inflammasome and caspase-1 cleavage (69). A truncated form of the IL-1 receptor, IL-1R3, is expressed primarily in the brain (70), although much is unknown about this receptor’s functions.

Following TBI, IL-1 is released early (hours-to-days), and can remain chronically elevated over levels seen in uninjured tissue (71, 72). Not only is IL-1 increased after TBI, but other molecules involved in the activation of the IL-1β pathway, including NLRP3, ASC, caspase-1, are also upregulated after TBI (73, 74). IL-1 is highly localized to the brain endothelium following a TBI (75). The time-course of post-TBI neuroinflammation has been heavily reviewed [see (5, 57, 76)], and it is not our goal here to re-do these wonderful reviews. Instead, we would like to demonstrate that IL-1 signaling is a common thread that can tie changes to the different CNS barriers post-TBI (**Figure 1C**).

**Figure 2:**
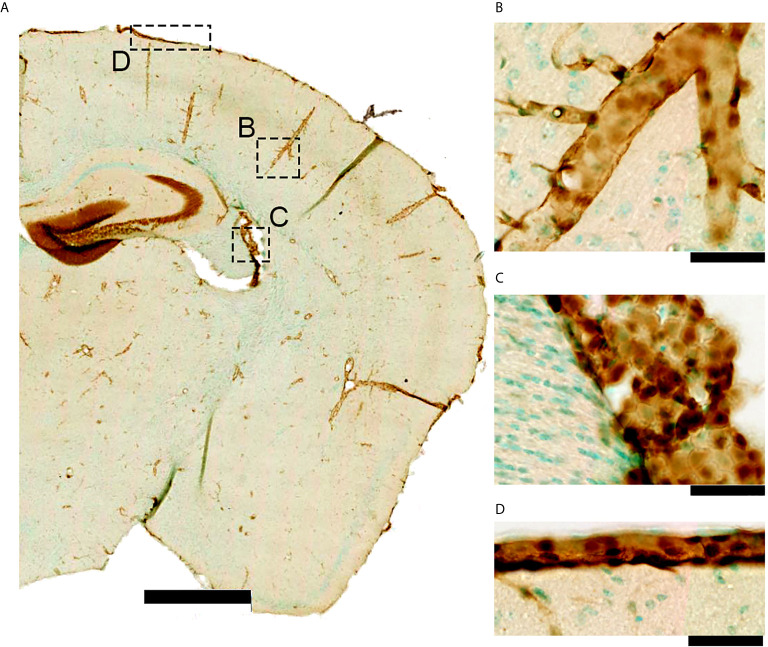
*IL-1R1* gene expression in a naïve adult reporter mouse. **(A)** The IL-1R1 reporter mouse line expresses RFP *via* the *IL-1R1* promoter (77) in discrete tissue compartments as seen using RFP immunohistochemistry. **(B)** IL-1R1 expression is high in blood vessels within the brain parenchyma, **(C)** choroid plexus, and **(D)** meninges. All of these areas are associated with one of the CNS barriers. (scale bar is 1mm in A, and 50 μm in **B–D**).

Leukocyte infiltration occurs mainly at the venous portion of the cerebral vasculature (60), where IL-1R1 expression is highest (**Figures 2A, B**). Leukocyte infiltration is a multi-step process, including rolling, activation, adhesion, and transmigration (78). IL-1 is responsible for activating endothelial cells as a part of the neuroinflammatory cascades resulting in increased expression of many major proteins involved in leukocyte adhesion and infiltration (79, 80). These IL-1 induced changes include alterations to junctional proteins [e.g., occludin and ZO-1 (81)], increased expression of vascular adhesion molecules [VCAM-1 and ICAM-1 *via* Nf-kB dependent mechanism (40, 79, 82)], and increase proinflammatory cytokines and chemokines [e.g., G-CSF, IP-10 and CCL2 (40)]. Further, pericytes can become activated by IL-1 signaling through IL-1R1 (83); however, a link with this mechanism in TBI has not yet been determined. IL-1R1 activation is thus sufficient for leukocyte infiltration across the brain endothelium (7), and leukocytes also release IL-1α and IL-1β (84), creating a positive feedback loop.

While a direct connection with IL-1 and post-TBI microbleeds has not been established, there is some correlative evidence suggesting a role of IL-1 in microbleeds. IL-1 signaling directly activates MMPs (85, 86). IL-1β and MMP-9 are correlated with an increased risk for the rupture of aneurysms (87) indicating a detrimental effect on the integrity of blood vessels in the brain. Further, IL-1 signaling stimulates the release of VEGF (23, 88), which increases CNS barriers permeability.

## Blood-Cerebrospinal Fluid Barrier

As early as 300 B.C., Roman physician Herophilos described a small structure of blood vessels found in the brain’s ventricles. Now, over 2000 years later, this structure known as the choroid plexus is still one of the most understudied structures in the CNS, despite its many significant roles in CNS functioning (89, 90). Namely, the choroid plexus is responsible for producing the cerebrospinal fluid (CSF) and contains the blood-cerebrospinal fluid barrier (89, 90). The blood-CSF barrier contains many mechanisms that create a separation between the sensitive CNS and the harsh peripheral environment.

### Blood-CSF Barrier Overview

**Figure 3:**
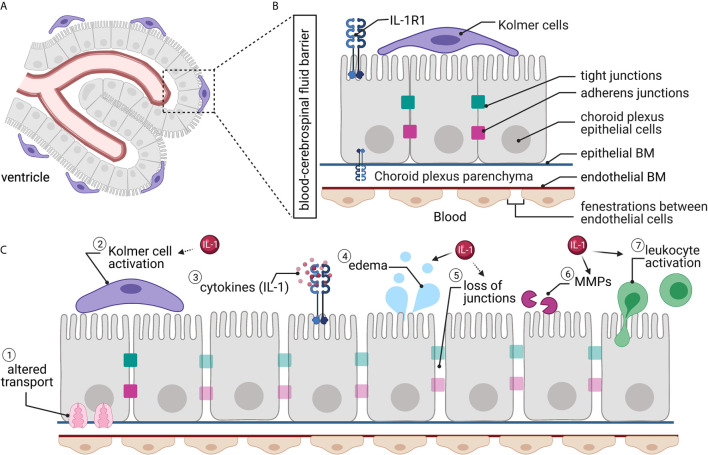
The blood-CSF barrier. **(A)** The blood-CSF barrier surrounds the choroid plexus situated within the ventricles of the brain. **(B)** The blood-CSF barrier’s endothelial cells are fenestrated, while the choroid plexus epithelial cells have tight junctions and adherens junctions between adjacent cells, creating a barrier. Kolmer cells interact with the choroid plexus epithelial cells on the ventricle side. Interleukin-1 receptor 1 (IL-1R1) is located on epithelial cells. **(C)** Traumatic brain injury causes alterations to the blood-CSF barrier, including (1) altered transporter localization and expression; (2) Kolmer cells become activated and retract their processes; (3) local and systemic increased cytokines; (4) increased water permeability and edema; (5) loss of tight junctions; (6) matrix metalloprotease (MMP) activity; and (7) increased leukocyte trafficking and activation. Many of these changes have been linked directly (solid arrow) or indirectly (dashed arrow) to IL-1 signaling.

The blood-CSF barrier consists of a single-cell layer of cuboidal epithelial cells that line and surround the highly vascular choroid plexus (91) (**Figure 3A**). The choroid plexus is responsible for producing CSF [for review, see (89, 90)]. The choroid plexus’s epithelial cells are the principal constituents of the blood-CSF barrier as well as the continuation of this barrier into the avascular arachnoid barrier, composed of the arachnoid mater and arachnoid villi of the meninges (12, 90, 92). The secretory function of the epithelium is reflected in their structure, as these cells have apical cilia that extend into the ventricles of the brain and serve to increase the surface area for secretion and monitoring of CSF composition (13, 91).

The choroid plexus capillary endothelial cells are uniquely fenestrated; thus, they are “leaky” (89–91, 93) (**Figure 3B**). The choroidal epithelial cells block paracellular diffusion of solutes into the ventricles *via* tight junctions and adherens junctions between cells (17). This serves to protect the CSF from changes in the blood milieu and maintain homeostasis (17). The blood-CSF barrier’s tight junctions contain the same families of proteins as those in the BBB but are considerably more complex. The proteins of the blood-CSF barrier tight junctions include claudins-1, -2, -3, and -11, occludin, and JAMs-A and -C (17). These proteins then bind to scaffolding proteins of the zona occludens family to link the barrier to the actin cytoskeleton (17). The adherens junctions of the blood-CSF barrier primarily contain E-cadherin, a transmembrane protein that binds to p120 and β-cantenin *via* its cytoplasmic tail (17). E-cadherin contributes to the barrier’s dynamic nature, as it regulates epithelial cell-cell contact and the plasticity of the junction (17).

While the tight junctions and adherens junctions tightly block paracellular diffusion, the epithelial cells, much like the endothelial cells of the BBB, have transporters that allow nutrients and other select molecules to cross into the CSF as well as to remove harmful substances (13, 19). The efflux of lipophilic solutes out of the epithelial cells and ventricles occurs *via* ABC family efflux pumps (18, 90). Conversely, a wide variety of solute-specific transporters manage the influx to the ventricles of ions and amino acids, as well as some other small hydrophilic molecules (18, 90). Aquaporin-1 is also present in the blood-CSF barrier and allows for the passive, osmotically-driven influx of water into the ventricles for CSF production (90).

The blood-CSF barrier draws similarities to the BBB in that it has associated cells that serve many roles in the optimal functioning of the barrier. In the blood-CSF barrier, these cells are a type of immune cell known as the Kolmer (or epiplexus) cells (91, 94). These choroid-associated macrophage-like cells were first described in 1921 by Kolmer (93, 94). The Kolmer cells attach to the microvilli on the apical side of the epithelial cells and serve as phagocytes and immune functions for the ventricular system (89, 95).

### Blood-CSF Barrier Disruption After TBI

The choroid plexus is highly sensitive to physical force and mechanical disruption caused by the primary injury after TBI (96). The third ventricle is particularly sensitive to frontal blows, while the lateral ventricles are more sensitive to lateral blows (97). Mechanical disruption of the choroid plexus can immediately alter fluid regulation in the brain resulting in exasperation of edema.

Traumatic brain injury causes several alterations to the blood-CSF barrier (**Figure 3C**). Damage to epithelial junctions can lead to increased permeability leading to altered CSF composition (97). Immediate loss of cilia on choroid plexus epithelial cells is also seen after TBI (96), reducing the ability to secrete CSF. The intracellular clefts in the blood-CSF barrier’s epithelial cell layer undergo swelling, and the clefts widen following experimental TBI (98, 99), increasing permeability through the blood-CSF barrier. VEGF and MMPs (specifically MMP-9) increase permeability and are elevated in the choroid plexus after CNS trauma (50, 100). Changes to the blood-CSF barrier can explain many TBI-induced metabolic changes. For example, a TBI induces ionic balance disruption, as transporters such as the Na-K-Cl transporter essential for restoring ion concentrations in the CSF and interstitial fluid, are reduced by the loss of ATP (101).

Intracranial pressure changes constitute a significant issue following TBI, leading to irreversible damage to the brain tissue (38). Edema results from increased fluid in the CNS parenchyma and is both a result of decreased efflux of fluid and increased influx (**Figure 3C**). The first phase of edema, termed vasogenic edema is due to the increased permeability of cells at the BBB, including endothelial cells and astrocytes (102–104). Vasogenic edema, characterized by the swelling of cells typically associated with the BBB, is also influenced by the blood-CSF barrier (101) and increases intracranial pressure. Reduced aquaporin-4, a water channel found on astrocytic end-feet, is linked with vasogenic edema in animal models as early as 24 hours after injury (105). The second phase of edema after TBI is cytotoxic edema. This phase is attributed to cell swelling within the brain parenchyma in the absence of BBB permeability changes (102–104) and lasts much longer after TBI (59).

Inflammation in the choroid plexus more closely resembles inflammatory processes in the periphery (93). Isolated choroid plexus express different cytokines and chemokines, including CXCL1, CXCL3, CCL2, LCN2, TNFα and IL-1β (57, 106, 107). Kolmer cells, the associated macrophage-like cell in the blood-CSF barrier, are altered after TBI, as seen by the appearance of short, blunt, cytoplasmic processes and the increase in vesicles (108) (**Figure 3C**). The reactive Kolmer cells appear to move toward the blood-CSF barrier’s damage, similar to functions of microglia and macrophages elsewhere in the brain (109).

Leukocyte transport from the periphery to the brain is also a primary physiological function of the choroid plexus (110). The choroid plexus expresses both P- and E-selectins, which are required for T cell recruitment (107, 110). Increased movement of leukocytes into the CSF has been found in experimental TBI (50, 64) (**Figure 3C**). Once inside the CSF, these cells infiltrate into the brain parenchyma. As with the BBB, most of the leukocytes infiltrating *via* the blood-CSF barrier are neutrophils (knowledge gained from multiple sclerosis models) (96, 111). These neutrophils are found to be associated with the choroid plexus and perivascular spaces.

### Mechanisms of IL-1 Signaling at the Blood-CSF Barrier After TBI

Most CNS studies of IL-1 signaling focus on the brain parenchyma; yet, there are essential functions for IL-1 pathway at the blood-CSF barrier (**Figure 3C**). IL-1 is released from choroid plexus cells epithelium (93). Because of the fenestrations within the capillaries in the blood-CSF barrier, this barrier is more sensitive to changes in the periphery. Specifically, systemic inflammation induces IL-1β expression in the choroid plexus (112). Further, IL-1R1 expression is high in the choroid plexus (113) (**Figure 2C**).

Brain injury in rodents results in increased IL-1β in the choroid plexus (107). IL-1 signaling in the blood-CSF barrier is correlated with increased permeability. Activation of enzymes, like MMPs, increases permeability by a breakdown of basal lamina and junctional proteins. Tissue inhibitors of metalloproteinases (TIMPs) are also found in high concentration in the choroid plexus (93), where IL-1 signaling is high.

IL-1 increases chemokine release (114), responsible for the infiltration of cells into the CSF and then subsequently moving into the CNS parenchyma. While Kolmer cells could engage in the trafficking of neutrophils (66), not much is known about the involvement of IL-1 in Kolmer cells function and activation, especially after TBI.

Loss of IL-1R1 in a hypoxia model reduced edema up to 90% compared to wildtype animals (115). Neutralizing antibodies for IL-1β delivered after TBI resulted in improved edema following mouse CCI (116). Yet, conflicting evidence suggest that genetic deletion of IL-1R1 was not successful at reducing brain edema after CCI (75). A complete mechanism for how IL-1 may be driving edema following TBI is not yet worked out, but likely involves the choroid plexus and the blood-CSF barrier.

## Meningeal Barrier

**Figure 4:**
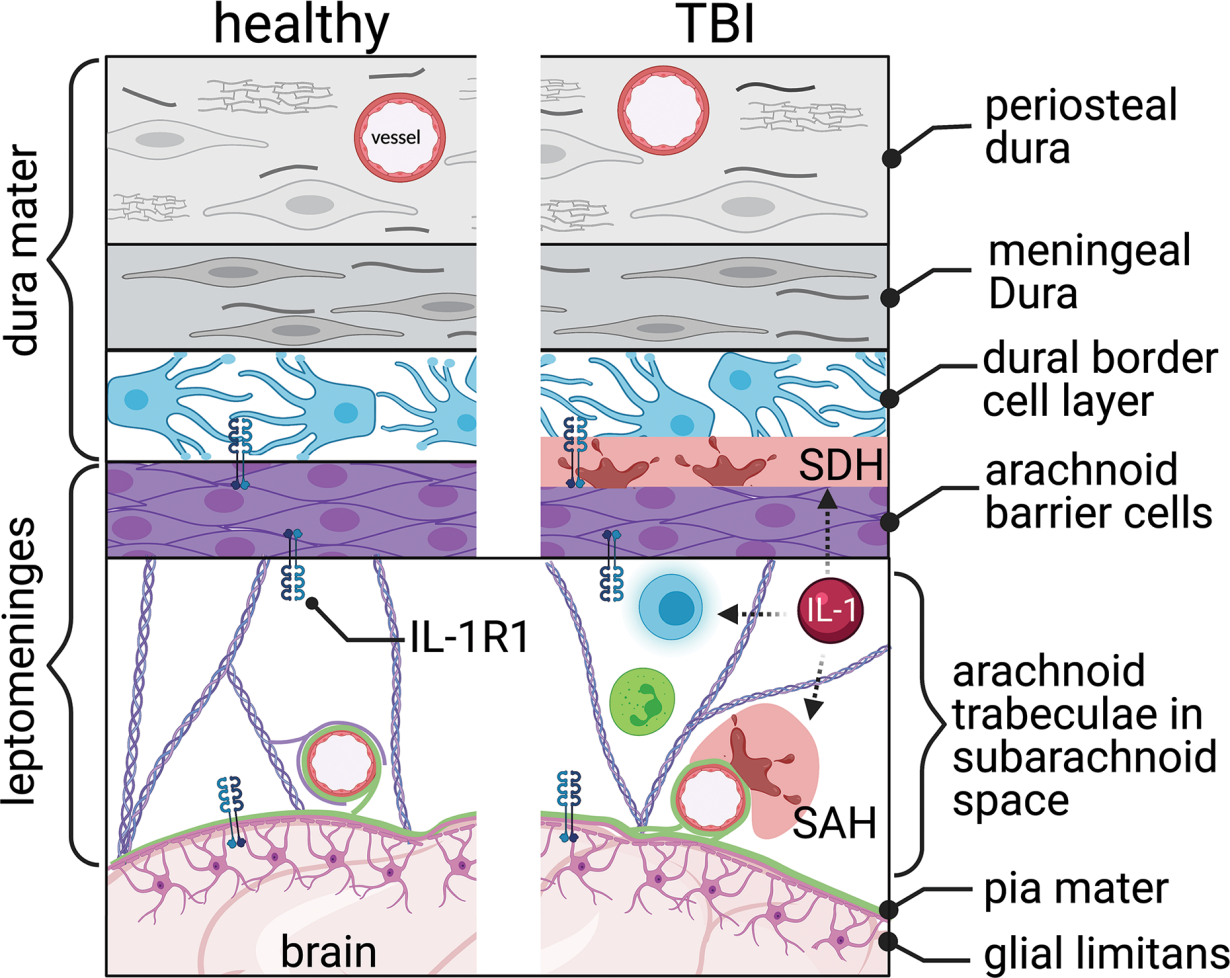
The meningeal barrier. The meninges, covering the brain surface, consist of three main layers, the dura mater, the arachnoid mater, and the pia mater. The dura mater consists of the periosteal, meningeal, and dural border cell layers. The arachnoid trabeculae imparts the majority of the barrier properties. The pia mater covers the brain’s surface and connects with the astrocytic end-feet’s glial limitans. Interleukin-1 receptor 1 (IL-1R1) is located within the arachnoid and pia mater. Traumatic brain injury tears the blood vessels running through and covered by the meninges, resulting in subdural hemorrhage (SDH) and subarachnoid hemorrhage (SAH). Increased immune cell trafficking occurs through the altered meninges post-TBI. IL-1 signaling can be linked indirectly (dashed arrows) with hemorrhage and leukocyte trafficking and activation.

The meninges are a grouping of different layers responsible for protecting and separating the CNS from the skull and periphery. There are three layers of meninges; dura mater, arachnoid mater, and pia mater, from outermost to innermost (**Figure 4**). The blood-CSF barrier is interconnected with the meningeal layer, specifically the arachnoid layer. Under homeostatic conditions, the meninges house many different local immune cell populations such as macrophages, dendritic cells, innate lymphoid cells, mast cells, neutrophils, B and T cells (117). Each meningeal layer has a different cellular consistency, density, and role in providing the CNS with a barrier between the brain and skull.

### Meningeal Barrier Overview

The dura mater, the most superficial meninges layer, comprises three layers: the periosteal, meningeal, and border cell layers (**Figure 4**). The outermost layer, periosteal dura, lines the inside of the skull and is composed of elongated fibroblasts (118, 119). The middle layer, meningeal dura, is made of flattened, elongated fibroblasts with small nuclei and dark cytoplasm (119). The innermost layer, the dural border cellular layer, is typically referred to as the subdural space or subdural compartment. This layer is structurally weak because of the flattened fibroblasts, which contain sinuous processes but lack collagen and elastic fibers (120).

The arachnoid mater lies directly below the dura mater (119). This meningeal layer is composed of two separate layers, each with its own function: arachnoid barrier cellular layer and arachnoid trabeculae (**Figure 4**). The arachnoid layer constitutes the majority of the meninges’ barrier function as an extension of the blood-CSF barrier. The many tight junctions within the arachnoid barrier cellular layer help divide the CSF-filled subarachnoid space from the fenestrated capillaries within the dura (121). The un-innervated and avascular arachnoid trabeculae consist primarily of connective tissue (119, 122). The sub-arachnoid space – lying below the arachnoid barrier cellular layer and within the arachnoid trabeculae – contains arachnoid trabeculae cells, CSF, arteries and veins, and cranial nerve roots (119).

The highly vascularized pia mater is tightly adhered to the brain’s surface, contouring to the gyri and fissures, and forms the leptomeninges with the arachnoid mater (**Figure 4**). The most superficial pial layer is composed of flat overlapping mesothelial cells, forming a tight connective layer, joined by desmosomes and gap junctions, rendering this layer impermeable to CSF along vessels within the perivascular space (121). Below this cellular layer, the epipial layer consists primarily of collagenous fibers, and the deeper intima pia is composed of reticular and elastic fibers (121). On the surface of the brain parenchyma, the glial limitans are formed by a dense network of astrocytic processes covered by an outer basal lamina interconnected with cells of the pia mater (123). Processes from surrounding pial cells envelop small vessels that penetrate the neural surface (119). These sheathed vessels create a perivascular space known as Virchow-Robins spaces (121).

### Meningeal Barrier and TBI

Trauma, especially to frontal regions of the head, can lead to CSF leakage, as dura adhered to the facial bones can be torn when facial bones fracture (124). CSF leaks can heal on their own in some cases. If left untreated, CSF leakages can lead to alterations in intracranial pressure, development of bacterial meningitis, or the formation of an intracranial abscess (124).

Vessels suspended in the meninges are particularly vulnerable to the mechanical strain of TBI. Trauma often causes bleeding in the vessels within and around the meninges. Blood pooled in the subdural space, known as subdural hematoma (120), is seen in 10-20% of TBI patients and is prevalent in severe TBI (125, 126) (**Figure 4**). Bleeding at the meningeal barrier following mild TBI is not well studied. Subdural hematoma is associated with increased mortality and poor outcomes (127). Chronic subdural hematoma may enlarge and compress nearby vasculature, causing increased intracranial pressure and compress neurological structures. Traumatic subarachnoid hematoma occurs when the mechanical force shear veins or arteries within the meninges (128) (**Figure 4**). Traumatic subarachnoid hematoma changes cerebral blood flow and cerebral vessels (129). MRI findings, using contrast fluid-attenuated inversion recovery (FLAIR), show that an increase in meningeal bleeding is seen following TBI and can be a useful diagnostic tool (128).

Following the initial impact and meningeal bleeding, a series of clotting and inflammatory responses are initiated (120). Many immune cells circulate through the meningeal barrier to act in a surveillance capacity. CD4 positive T cells traffic through and function to modulate the inflammatory make-up of the CSF, which in turn can influence neuronal function (130). In response to TBI, these T cells recognize danger-associated molecular patterns (DAMPs) released from the brain into the CSF. Damaged and necrotic cells caused by the initial trauma of TBI release DAMPs (130). These can be various molecules including IL-1α, IL-33, HMGB1, S100b, mitochondrial DNA, and ATP among others and are sensed by pattern recognition receptors (131). In CNS injury, this alarm signal induces chemokine secretion, which recruits peripheral immune cells, like monocytes and macrophage cells, into the brain *via* the meningeal blood vasculature (132). Lacking either the alarm signal (IL-33) or the chemokine receptor (CCR2) results in reduced infiltration of peripheral immune cells following neurotrauma (132).

### Mechanisms of IL-1 Signaling at the Meninges Post-TBI

Meninges are significantly enriched sources of IL-1β within the CNS (112, 133). IL-1R1 is also expressed within the meninges (113), and by the immune cells in the meninges (**Figure 2D**). Thus, the meningeal barrier is an active zone for IL-1 signaling. For instance, the presence of IL-1β in the meninges could cause local T cell activation and expansion following a TBI (134, 135); however, this remains to be experimentally defined. Indeed, there are significant gaps in knowledge regarding the function of IL-1/IL-1R1 in post-traumatic subdural or subarachnoid hematoma (**Figure 4**).

In models of subdural hematoma, there are an increased expression of inflammatory cytokines, neutrophil infiltration, and VEGF (136, 137). These changes suggest a possible role for IL-1/IL-1R1 in the secondary injury cascade of a subdural hematoma. Increased levels of the IL-1 family of proteins occur in people with subarachnoid hemorrhage and correlates with poorer outcomes (138, 139). Therapeutic administration of exogenous IL-1ra – to antagonize IL-1R1 – in patients with subarachnoid hematoma, reduced inflammation markers (IL-6 and C-reactive protein), and improved Glasgow Outcome Scale scores (140). More studies are needed to establish a causal role for IL-1/IL-1R1 signaling in the pathophysiology of traumatic subdural and subarachnoid hematomas.

## Blood-Retinal Barrier

**Figure 5:**
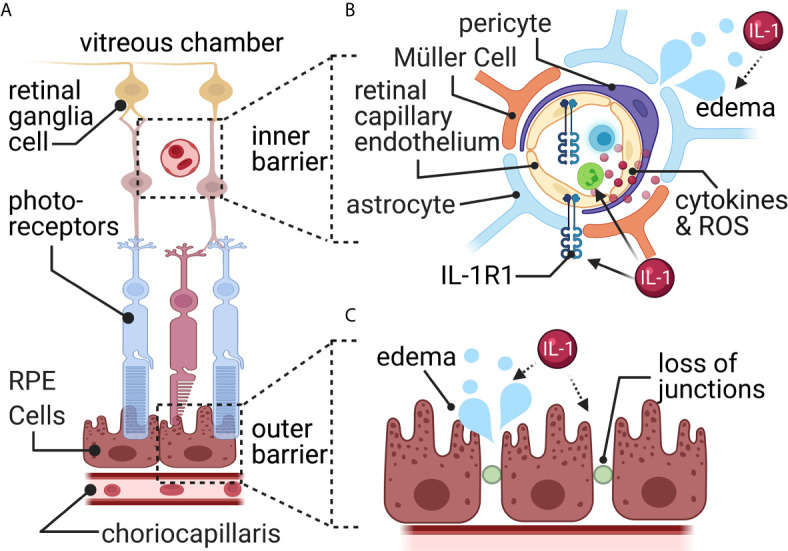
The blood-retina barrier. **(A)** The blood-retina barrier consists of two separate layers. The first (outer) around the choriocapillaris blood vessels, and the second (inner) around the blood vessels in the retinal ganglia cell layer. **(B)** The inner barrier consists of endothelial cells surrounded by pericytes, astrocytic end-feet, and specialized glial cells called Müller cells. Here, a TBI increases permeability and results in edema and increased leukocyte infiltration. Müller cells pull away from the barrier and activate to release reactive oxygen species (ROS) and cytokines. **(C)** The retinal pigment epithelial cells (RPE) connected *via* tight junctions form the outside barrier with the choriocapillaris blood vessels. Here, a TBI causes increased fluid accumulation through disrupted tight junctions resulting in edema. Most connection with IL-1 signaling is indirect (dashed arrows), while some direct (solid arrow) evidence connects IL-1 signaling with leukocyte trafficking.

Characterized over 100 years ago by Schnaudigel, the blood-retinal barrier provides an integral boundary between circulation and the retina necessary to maintain a homeostatic environment. The blood-retinal barrier is separated into an inner and outer barrier, each with a distinct structure, leading to a complete barrier (**Figure 5A**).

### Blood-Retinal Barrier Overview

The inner blood-retinal barrier is located within the retinal microvasculature and resembles the BBB (**Figure 5B**). It contains tight junctions between retinal capillary endothelial cells and is surrounded by pericytes, Müller cells, and astrocytes (141). The Müller cell is responsible for forming and maintaining the tight junctions between glia and endothelial cells (142, 143). Astrocytes also provide various functions similar to the Müller cell, such as structural support, forming and maintaining tight junctions, and forming the blood-retinal barrier (144, 145). Pericytes, located on the surface of retinal blood vessels, are also critical in maintaining the blood-retinal barrier *via* regulating blood flow and angiogenesis (146).

The outer blood-retinal barrier is comprised of tight junctions between adjacent retinal pigment epithelium (147) and resembles the structure of the blood-CSF barrier (**Figure 5C**). The outer blood-retinal barrier separates the neural retina from a network of vessels known as the choriocapillaris – the large blood supplier for the neural retina (148). Tight junctions allow the outer blood-retinal barrier to monitor and filter the influx of solutes and nutrients from the choroid to the sub-retinal space (147).

Paracellular transport across the blood-retinal barrier is tightly regulated and is impermeable to larger molecules such as glucose, amino acids, etc. (148, 149). However, the retina is one of the most metabolically active tissues (150, 151). It contains high concentrations of glucose transporters (152–154), and lactate transporters (155–157). For a review of specific influx and efflux transporter, see (158).

### Blood-Retinal Barrier and TBI

People often report increased sensitivity to light, retinopathy, optic neuropathy, dysfunctional optic motility, and visual field deficits following a TBI (159). Of the millions living with long-term TBI-induced disabilities, 20-40% have visual issues (160), and prevalence can be higher in specific populations, such as the military (161).

While many TBI-induced visual deficits originate within the brain stem and visual cortex, significant changes occur to the visual system’s peripheral portions, including the blood-retinal barrier (162). The retinal ganglion cells – responsible for sensation of light and the formation of the optic nerve – are linked to common post-TBI symptoms including dry eye, headache, and sleep issues (163). TBI causes cell death and thinning of the retinal ganglion cell layer, including apoptosis of the retinal ganglion cells (160, 164, 165). Retinal ganglion cells are susceptible to reactive oxygen species, such as that found in the retina after a CCI in mice (160) (**Figure 5B**).

Macular edema, which involves fluid accumulation within the retinal layers that causes reduced blood flow and visual impairments, is often seen following trauma (149). Multiple cell types are responsible for the increased blood-retinal barrier permeability and fluid disruptions. Müller cells release MMPs, breaking down tight junctions within the inner and outer barriers (149), however a connection with TBI has not yet been discovered. Leukocytes, adhered to retinal vessels, produce nitric oxide and other pro-inflammatory mediators, increasing the blood-retinal barrier’s leakiness after a TBI (149) (**Figure 5B**). Retinal pericytes’ function post-TBI remains undefined. Pericytes changes, such as in diabetic retinopathy, can alter vascular tone, and cause edema and ischemia (149, 166).

### Mechanisms of IL-1 Signaling at the Blood-Retinal Barrier Post-TBI

IL-1β is increased in the optic nerve (167), and blood-retinal barrier (168) and IL-1R1 is increased in the retina (169) in experimental models of TBI. The exact source of IL-1 in the retina has not been worked out. The IL-1 could signal *via* the blood-retinal barrier’s endothelial cells that express IL-1R1 (169). Retinal pigment epithelium exposed to IL-1β, *in vitro*, reduces claudin-1, ZO-1, and occludin (170, 171). *In vitro*, retinal pericytes release inflammatory mediators (such as, IL-8, CCL2 and CCL5), and increase expression of adhesion molecules (such as, ICAM-1 and VCAM-1), following IL-1β stimulation (172). *In vivo*, direct stimulation with IL-1β causes increased leakiness of the endothelial cell barrier (173). Thus, IL-1 could potentially be a mechanism for increased permeability and leukocyte trafficking at the blood-retinal barrier after TBI; however, experimental confirmation in TBI models is lacking (**Figures 5B, C**).

## Barrier of the Lesion Core

Astrocytes and meningeal fibroblasts form the lesion barrier or ‘scar’ after a TBI (174, 175). The astrocytic barrier closes the wound and restricts leukocyte trafficking, protecting the remaining neurons (23). Extracellular matrix proteins like collagen, laminin, and fibronectin, secreted by fibroblasts, are further deposited in the lesion barrier (175). Leukocytes infiltrate within the fibrotic scar and contribute to debris clearance and inflammation (175). While thought to be primarily protective, the lesion barrier’s scar is also associated with increased inflammation, neuronal damage, post-traumatic epilepsy, BBB disruption, and tissue damage (176, 177).

### Mechanisms of IL-1 Signaling at the Lesion Core After TBI

The astrogliosis required for the formation of the astrocyte scar at the lesion following TBI can be directly activated *via* IL-1 released mainly through microglia. In mice lacking IL-1R1, GFAP immunoreactivity was delayed following a penetrating TBI model (178). *In vitro*, astrocytes exposed to IL-1β increase levels of GFAP, a characteristic response of astrogliosis (179). *In vitro*, IL-1 exposure increases fibroblast production of extracellular matrix proteins (180, 181). More work is warranted to directly test astrocyte and fibroblast IL-1 signaling in scar formation after TBI.

## Conclusion

TBI causes a plethora of pathologies to the CNS barriers, including but not limited to the BBB. Each CNS barrier is unique, yet most mechanistic understanding of the TBI-induced barrier pathophysiology is limited to the BBB. Common among all the barriers are increased inflammatory reactions and increased immune cell trafficking from the periphery into the CNS. CNS barriers express IL-1R1 (**Figure 2**), and IL-1 signaling induces leukocyte infiltration (7, 182, 183) and many other inflammatory-associated barrier changes. IL-1 is associated with worse outcomes in patients with TBI (74, 184). Further, IL-1 is associated with an increased risk for developing post-traumatic epilepsy (185, 186), and blocking IL-1 signaling reduces lesion size, neurodegeneration, and improves behavior (187–189). For review of IL-1 therapies see (190, 191).

Many of the current connections between IL-1 signaling post-TBI are correlative. The BBB and blood-CSF barrier provides the most comprehensive of the TBI and IL-1 interactions. However, a direct connection between IL-1 signaling and several post-traumatic barrier pathologies, including microbleeds, pericyte disruption, loss of junctional proteins, Kolmer cells at the blood-CSF barrier, all require further investigation. The meningeal barrier and the blood-retinal barrier present the most opportunity for further exploration in the TBI field as there is no knowledge of IL-1’s function in these barriers. Most work at the CNS barriers has focused on IL-1β; thus, some assumptions remain regarding the function of IL-1α at the CNS barriers. Future research is needed to determine differential effects of IL-1α and IL-1β at the CNS barriers, as IL-1α and IL-1β can cause differing responses (192, 193). As scientific tools become more advanced, the understanding of IL-1’s role in TBI-induced barrier dysfunction will improve, as IL-1 is an attractive target for CNS barrier targeted interventions.

## Author Contributions

CB, JW, and EH completed the literature review and wrote the first draft. NQ contributed to the design and conceptualization. AB contributed to the design and conceptualization, wrote and compiled sections, and revised the manuscript for intellectual content. All authors contributed to the article and approved the submitted version.

## Funding

This publication was supported in part by National Institutes of Health under award numbers F31NS116912, R21AG066865, R21AG059123, R01NS103785, 5T32NS077889-08, and the Department of Defense award number AZ190017.

## Disclaimer

The content is solely the responsibility of the authors and does not represent the official views of the National Institutes of Health or the Department of Defense.

## Conflict of Interest

The authors declare that the research was conducted in the absence of any commercial or financial relationships that could be construed as a potential conflict of interest.
